# Erratum to: Development and feasibility study of very brief interventions for physical activity in primary care

**DOI:** 10.1186/s12889-015-2459-x

**Published:** 2015-11-17

**Authors:** Sally Pears, Katie Morton, Maaike Bijker, Stephen Sutton, Wendy Hardeman, on behalf of the VBI Programme Team

**Affiliations:** Behavioural Science Group, Primary Care Unit, Institute of Public Health, Forvie Site, University of Cambridge, School of Clinical Medicine, Cambridge Biomedical Campus, Cambridge, CB2 0SR UK

Upon publication of this article [1] it has been noted that due to a technical error the publisher missed to include the Open Access Licence statement in the copyright line. The correct copyright line should have read:

© 2015 Pears et al. This is an Open Access article distributed under the terms of the Creative Commons Attribution License (http://creativecommons.org/licenses/by/4.0), which permits unrestricted use, distribution, and reproduction in any medium, provided the original work is properly credited. The Creative Commons Public Domain Dedication waiver (http://creativecommons.org/publicdomain/zero/1.0/) applies to the data made available in this article, unless otherwise stated.
